# A general method to eliminate laboratory induced recombinants during massive, parallel sequencing of cDNA library

**DOI:** 10.1186/s12985-015-0280-x

**Published:** 2015-04-09

**Authors:** Caryll Waugh, Deborah Cromer, Andrew Grimm, Abha Chopra, Simon Mallal, Miles Davenport, Johnson Mak

**Affiliations:** School of Medicine, Deakin University and CSIRO(AAHL), Geelong, VIC Australia; Biosecurity Flagship, CSIRO(AAHL), Geelong, VIC Australia; Centre for Vascular Research, University of NSW, Sydney, NSW Australia; Institute for Immunology and infectious diseases (IIID), Murdoch University, Perth, WA Australia

**Keywords:** HIV, Recombination rate, Mutation rate, Transfection

## Abstract

**Background:**

Massive, parallel sequencing is a potent tool for dissecting the regulation of biological processes by revealing the dynamics of the cellular RNA profile under different conditions. Similarly, massive, parallel sequencing can be used to reveal the complexity of viral quasispecies that are often found in the RNA virus infected host. However, the production of cDNA libraries for next-generation sequencing (NGS) necessitates the reverse transcription of RNA into cDNA and the amplification of the cDNA template using PCR, which may introduce artefact in the form of phantom nucleic acids species that can bias the composition and interpretation of original RNA profiles.

**Method:**

Using HIV as a model we have characterised the major sources of error during the conversion of viral RNA to cDNA, namely excess RNA template and the RNaseH activity of the polymerase enzyme, reverse transcriptase. In addition we have analysed the effect of PCR cycle on detection of recombinants and assessed the contribution of transfection of highly similar plasmid DNA to the formation of recombinant species during the production of our control viruses.

**Results:**

We have identified RNA template concentrations, RNaseH activity of reverse transcriptase, and PCR conditions as key parameters that must be carefully optimised to minimise chimeric artefacts.

**Conclusions:**

Using our optimised RT-PCR conditions, in combination with our modified PCR amplification procedure, we have developed a reliable technique for accurate determination of RNA species using NGS technology.

## Background

The massive capacity of next generation sequencing (NGS) technologies is revolutionising transcriptome analysis and can be used to assess the complexity of viral diversity in highly mutable, genomes of RNA viruses that cause disease in humans, such as human immunodeficiency virus-1 (HIV-1). Accurate detection of clinically relevant, drug-resistant mutations is important for adapting patient drug regimes. In addition, deep sequencing is used to investigate host cell and viral factors that impact on the genetic diversity of HIV-1.

The production of cDNA libraries for NGS involves laboratory procedures that include RNA extraction, reverse transcription PCR (RT-PCR) to generate cDNA followed by PCR amplification. Importantly, the amplification of each cDNA species must remain clonal, especially when studying the diversity of RNA species. The laboratory manipulations necessary for library generation are recognised as potential sources of artefactual recombination. Several papers have addressed the issue of minimising PCR artefacts [1-3] and there is growing interest in optimising RT-PCR techniques to enable accurate analysis of patient RNA virus load and early detection of the emergence of drug resistant quasi-species [3-6].

Thus, in both the clinical and experimental setting there is a recognised need to identify sources of laboratory induced error and to minimise these to obtain a robust technique for generating DNA libraries for NGS that faithfully reflect the underlying genetic diversity of the virus. In the current study there were three aspects of sample processing that we were particularly interested in assessing and optimising: RNA concentration during first strand synthesis; effect of RNase H activity and PCR cycling conditions. To assess the impact of these on the introduction of recombinant artefact we used a mix of authentic wild-type (WT) HIV-1 and marker virus containing silent mutations[7] that are ideal for assessing sources of laboratory induced recombination events during cDNA library preparation. The marker virus is biologically and functionally indistinguishable from the parental WT virus, and thus mimics the closely related, yet genetically distinct, quasi-species that exist in infected patients.

Using our viral system we have produced populations of genetically distinct, but functionally identical, homozygous HIV-1 particles (containing either WT or marker RNA) and also heterozygous HIV-1 (containing a strand of WT and marker RNA) as sources of viral RNA species for analysis of laboratory sources of recombination. We have characterised the important sources of error in RT-PCR that contribute to artificial chimera formation and, in combination with our modified PCR amplification procedure [1] and analytic tools [7], have developed a reliable technique for accurate determination of viral diversity using NGS. Our key observations are that (i) the amount of RNA used in the reverse transcription PCR must be kept to a minimum; (ii) approximately 2,500 copies of cDNA should be input into the PCR amplification step and (iii) PCR cycles should be optimised so that the amplification reaction is stopped whilst still in the linear phase (27–29 cycles). Furthermore, we have also shown that homologous recombination during transfection, sometimes perceived as a source of experimental bias during virus production [8-11], is a negligible source of error and the effect of laboratory manipulations on estimates of mutation rate can be readily managed with appropriate rigorous experimental procedure.

## Results and discussion

### Increasing RNA concentration correlates with artificial chimera formation in reverse transcription PCR (RT-PCR)

**Figure 1:**
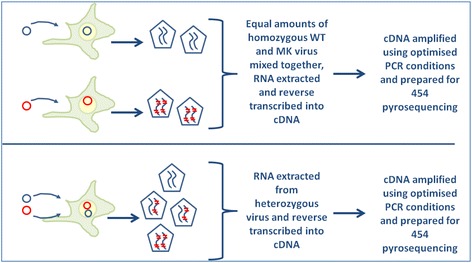
**Experimental method for assessing transfection as a source of artificial chimeras.** Virus was produced by transfection of HEK293T cells, harvested and quantitated using an ELISA assay to detect HIV-1 p24^CA^ protein. In these studies RNA was extracted from either a mix of equal amounts of homozygous WT and homozygous MK virus (top) or from an equivalent amount of heterozygous virus (bottom).

Many groups have exploited the depth and sensitivity of NGS to characterise viral quasispecies in patient samples especially to identify rare HIV-1 variants and clinically important drug resistant mutations [3-6]. Where plasma or tissue viral load is low, it is imperative that efforts to maximise cDNA product for analysis, such as increasing (a) the amount of RNA that is reverse transcribed or (b) the amplification cycles during PCR, do not compromise the original virus population species. To estimate the effect of initial RNA concentration on the detection of chimeric recombinants an equal amount (based on viral protein p24^CA^) of homozygous WT and MK viruses were mixed together and RNA extracted (Figure 1 top). First strand synthesis was performed with three different concentrations of starting material using the high fidelity SSIII RT. RNA was added to the reverse transcription reaction mix at 160 ng, 1600 ng or 3990 ng, the maximum amount possible in this experiment given the concentration of the extracted RNA. It should be noted that these amounts are within the range recommended by the manufacturer (1 pg – 5ug). In order to stress the RT-PCR conditions and to mimic the effect of excessive template, at the highest template concentration (3990 ng), gene specific primers and dNTP’s were reduced 4-fold.

**Figure 2:**
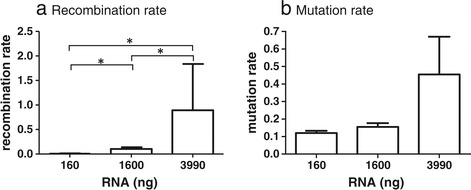
**Effect of RNA concentration on (a) recombination and (b) mutation rates.** RNA was extracted from an equal mix of WT and MK virus. SSIII was used to reverse transcribe 160 ng, 1600 ng or 3990 ng as input template. At the highest RNA concentration dNTP’s and primers were reduced 4-fold to mimic the effect of excess RNA template. Resulting cDNA was diluted such that ~2500 copies were input and amplified using an optimised 2-step PCR and reduced (27) cycles to minimise PCR-induced recombination [1]. Recombination and mutation rates are expressed as rate per 1000 nucleotides and the confidence intervals, as estimated via bootstrapping, are shown. Significant differences (at 95%CI) are indicated.

The recombination events per 1000 nucleotides (REPN) increased significantly as RNA concentration increased for RNA extracted from the mix of WT and MK virus (Table 1a and Figure 2a). In addition, the number of relevant sequences retrieved from 454 sequencing declined as the RNA input template increased and notably, the greatest proportion of truncated sequences and the lowest proportion of sequences suitable for analysis occurred at the highest input template (data not shown). There was a less pronounced effect on mutation with similar rates for low and mid-range input RNA templates, rising approximately 3-fold at the highest input template (Table 1b and Figure 2b). These data indicate that the concentration of RNA during reverse transcription can have a profound effect on the quantity and quality of sequencing data, can promote formation of artificial chimeras but has less impact on mutation rate.

**Table 1:** **Recombination and mutation rates**

**a. Recombination rates**						
**RNA (ng)**	**RT**	**Virus**	**PCR Cycles**	**RR**	**Lower 95% CI bound**	**Upper 95% CI bound**
160	SSIII	wt + mk mix	27	0.0065	0.0000	0.0132
1600	SSIII	wt + mk mix	27	0.1029	0.0787	0.1391
3990	SSIII	wt + mk mix	27	0.8933	0.3675	1.8378
2000	AMV	wt + mk mix	27	0.0742	0.0640	0.0901
160	SSIII	Heterozygous	27	0.0128	0.0064	0.0248
160	SSIII	wt + mk mix	35	2.9482	2.7699	3.1668
1600	SSIII	wt + mk mix	35	4.6068	4.4601	4.7671
3990	SSIII	wt + mk mix	35	1.061	0.7903	1.4239
**b. Mutation Rates**						
**RNA (ng)**	**RT**	**Virus**	**PCR Cycles**	**MR**	**Lower 95% CI bound**	**Upper 95% CI bound**
160	SSIII	wt + mk mix	27	0.1198	0.1075	0.1325
1600	SSIII	wt + mk mix	27	0.1551	0.1340	0.1763
3990	SSIII	wt + mk mix	27	0.4549	0.2674	0.6702
2000	AMV	wt + mk mix	27	0.2169	0.2053	0.2288
160	SSIII	Heterozygous	27	0.1658	0.1503	0.1811
160	SSIII	wt + mk mix	35	0.2172	0.1959	0.2396
1600	SSIII	wt + mk mix	35	0.1608	0.1494	0.1717
3990	SSIII	wt + mk mix	35	0.2819	0.2483	0.3166

RNA was extracted from either an equal mix of homozygous WT and MK viruses or from an equivalent amount of heterozygous virus and reverse transcribed using increasing amounts of RNA as template using SSIII reverse transcriptase (RT), engineered to have minimal RNase H activity. In a parallel experiment RNA was also reverse transcribed using AMV with intact RNase H activity. Following first strand synthesis, cDNA was diluted as appropriate and amplified using 27 or 35 PCR cycles. Replicates containing SYBR Green 1 dye were used to ensure the reaction was stopped while in the log linear phase (27 cycles) or at plateau (35 cycles). Replicate samples were pooled and prepared for 454 NGS sequencing. Recombination and mutation rates are expressed as rate per 1000 nucleotides. The 95% confidence interval lower and upper boundaries, as determined by bootstrapping, are shown for each estimate.

### Effect of PCR cycling conditions on chimera formation

**Figure 3:**
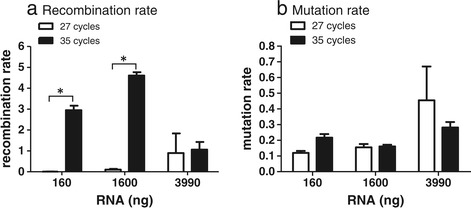
**Effect of PCR cycle on (a) recombination and (b) mutation rates.** RNA was extracted and transcribed as described in Figure 2. Resulting cDNA was diluted as described and amplified using a 2-step PCR and either 27 cycles (white bars) or 35 cycles (black bars). Recombination and mutation rates are expressed as rate per 1000 nucleotides and the confidence intervals, as estimated via bootstrapping, are shown. Significant differences (at 95%CI) are indicated.

It has been shown that PCR conditions have a significant impact on the detection of genomic recombinants [1]. Smyth et al. have developed modified cycling conditions to minimise the occurrence of chimeras during PCR. We sought to estimate the cumulative effects of optimal versus non-optimal RT-PCR followed by optimal and non-optimal PCR conditions. RNA was reverse transcribed, as described, varying the input amounts in the RT-PCR and then diluted aliquots of cDNA were amplified with PCR using identical conditions other than the number of amplification cycles. PCR amplification was performed with optimised cycles, where the amplification process was terminated in the linear phase (25–28 cycles), or allowing the PCR to run to plateau (35 cycles). Amplification was followed in real time by monitoring duplicate samples that contained the fluorescent dye SYBR Green 1. As expected, allowing the PCR amplification to run to ‘completion’ resulted in significant increases in recombination rate in all cases (Table 1a compare 27 cycles to 35 cycles and Figure 3a). The number of PCR cycles had very little impact on mutation rate (Table 1b and Figure 3b).

### RNAse H activity has minor impact on chimera formation

The HIV-1 genome is comprised of 2 identical (or near identical) strands of RNA, that are used as template during reverse transcription to produce a cDNA molecule for integration into the host genome during infection. Template switching during reverse transcription is acknowledged as an efficient mediator of viral diversity, generating recombinant HIV RNA species that are a mixture of the two parental RNA strands. Template switching is thought to be facilitated through the action of the RNase H activity of the HIV-1 RT whereby a dynamic balance exists between the polymerase activity of RT and the endonuclease activity of RNase H that cleaves the RNA from the nascent cDNA polymer [12,13]. According to the copy-choice model, RNase H degrades the RNA template from the RNA/cDNA hybrid thus permitting the newly synthesised DNA to base-pair with the similar sequence on the other RNA strand.

**Figure 4:**
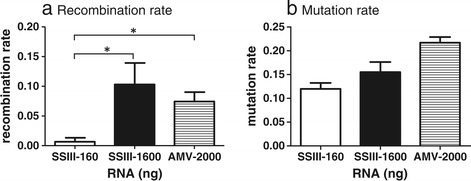
**Effect of RNase H activity on (a) recombination and (b) mutation.** RNA was extracted from an equal mix of WT and MK virus. SSIII was used to reverse transcribe 160 ng and 1600 ng as input template and AMV was used to reverse transcribe 2000 ng. All conditions complied with the manufacturers recommendations. Resulting cDNA was diluted and amplified using an optimised 2-step PCR and 27 cycles to minimise PCR-induced recombination [1]. Recombination and mutation rates are expressed as rate per 1000 nucleotides and the confidence intervals, as estimated via bootstrapping, are shown. Significant differences (at 95% CI) are indicated.

As the aim of this study was to identify optimal processes for minimising laboratory induced artefact we performed a comparative analysis of AMV with SSIII. To assess the *in vitro* effect of RNase H activity on the formation of artificial recombinants viral RNA was reverse transcribed using either the high fidelity SSIII enzyme, engineered to have reduced RNase H activity, or AMV RT with full RNase H activity. We compared the recombination rates detected for 160 ng and 1600 ng of RNA transcribed with SSIII with the rate for 2ug RNA transcribed using AMV-RT, as recommended by the manufacturer. Surprisingly, the RNase H activity of AMV had little impact on the recombination rate when compared to 1600 ng of RNA transcribed with SSIII, however both of these were 10-fold higher than the recombination rate at 160 ng input RNA using SSIII (Table 1 and Figure 4a). These results further emphasise the influence of RNA concentration on recombination during first strand synthesis. The mutation rate was similar for both enzymes (Table 1 and Figure 4b) and, in contrast to the recombination rate, was not significantly affected by RNA concentration.

### Transfection of HEK293T cells does not contribute significantly to chimera formation

Several groups have developed experimental protocols to measure recombination rates of HIV-1 and other viruses that require the production of virus from producer cell lines using standard transfection methods and then use of the virus to infect cell lines and primary cells [14-17]. Thus, transfection presents another potential source of artificial recombination. Indeed, previous studies have shown that mammalian cells possess the enzymatic machinery to effect homologous recombination and that (i) DNA topology affects homologous recombination with linear molecules the preferred substrates [9], (ii) homologous recombination can occur between single stranded and double stranded DNA [10] and (iii) nicked DNA significantly enhances the frequency of homologous recombination in transfected cells [8,11]. Our system of marker and wild type plasmids provides a unique opportunity to assess the role of homologous recombination as a source of chimeric artefact during transfection. HEK293T cells were transfected with proviral DNA plasmids to produce control viruses. We extracted RNA from either an equal mixture of homozygous WT and homozygous MK viruses (Figure 1 top) or from heterozygous WT/MK virus (based on p24 values) (Figure 1 bottom). Our sequencing data reveals that the heterozygous virus is composed of ~50% WT: 50%MK HIV-1 sequences (data not shown). This is consistent with the premise that the plasmids used in transfection were equally transcribed. Given that: (i) the WT and MK plasmids were transfected in equal ratios, (ii) each virion contains two copies of RNA and (iii) the heterozygous virus also has equal ratios of WT and MK virus, then the simplest and most likely scenario is that 25% of virions contain two copies of WT virus, 25% of virions contain two copies of MK virus and 50% of virions contain one copy of WT virus and one copy of MK virus. Any other scenario is less likely and would require special pleading, and therefore we believe that our Mendelian assumption is valid. This is also consistent with data published by Chen et al. [18] who were able to directly discriminate between two different RNA molecules encapsidated within the same virion particle and confirmed that, using standard transfection techniques, RNA co-packaging was an efficient process (more than 90% of virions contained RNA) and that ~48% of virions that contained RNA were heterozygous, as predicted by the Hardy-Weinberg model.

Samples were prepared using optimised RT-PCR (160 ng input RNA template) and resulting cDNA was diluted so that approximately 2,500 copies of HIV-1 cDNA were used as template and amplified using our optimised PCR protocols. The mixture of homozygous RNA species provides a baseline measure of artefactual recombination attributable to downstream laboratory manipulations, as any recombination between these homozygous viruses could only have occurred during RT-PCR, PCR or 454 sequencing. Thus, comparison of the rate of recombination between the mix of homozygous viruses (equal mix of WT plus MK) with that of the truly heterozygous viral RNA provides a novel and accurate estimate of the rate of transfection-induced recombination.

**Figure 5:**
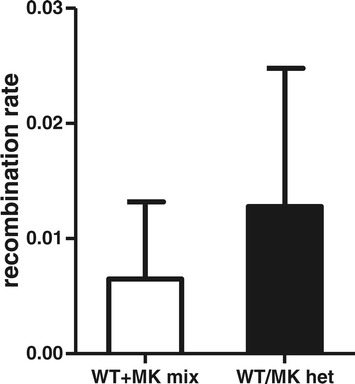
**Occurrence of transfection induced recombination.** Homozygous and heterozygous viruses were produced by transfection of HEK293T cells as described in the methods. RNA was extracted from an equal mix of WT and MK virus or from an equivalent amount of heterozygous virus. In parallel reactions SSIII was used to reverse transcribe 160 ng as input template. Resulting cDNA was diluted and amplified using an optimised 2-step PCR and 27 cycles to minimise PCR-induced recombination [1]. Any recombination occurring in the mix of homozygous viruses can only be a consequence of RT-PCR, PCR and/or 454 sequencing (laboratory induced recombination). Recombination in the heterozygous virus reflects both laboratory induced recombination and transfection induced recombination. Recombination and mutation rates are expressed as rate per 1000 nucleotides and the confidence intervals as estimated via bootstrapping are shown. There was no significant difference between these estimates.

Table 1 and Figure 5 show that the detection of recombinant species in the heterozygous virus was extremely low and less than 2-fold higher than the mix of viruses. These results are consistent with those of Levy et al. [14] who used a GFP reporter system and found that recombination within transfected cells to generate heterozygous viruses resulted in approximately 0.33% of the producer cells exhibiting GFP fluorescence. Taken together, the data indicate that the predominant sources of artefactual recombination are the RT-PCR and PCR steps and that increasing input RNA in the RT-PCR step and using sub-optimal PCR conditions contribute significantly to the formation of chimeric species detectable by deep sequencing.

## Conclusions

Any analysis of viral diversity through experimentation raises the possibility that recombinant quasi-species could arise as a result of laboratory manipulations, including virus production (transfection), infection of primary cells or cell lines, RNA extraction and conversion of RNA to cDNA (RT-PCR) and cDNA amplification. Mutations in the form of insertions, deletions or substitutions could occur during any of these procedures and deep sequencing. Further, with the vast improvements in treatment for HIV-1 infection that have occurred over the last 30 years, clinicians are now able to investigate the emergence of viral quasi-species in individual patients and to tailor drug regimes to counter the emergence of deleterious, drug-resistant mutants. Experimental programs to identify viral and host cell factors that affect recombination and clinical assessments of drug-resistant mutants require sensitive and validated methods to discern ‘true’ *in vivo* viral species from laboratory induced artefacts.

We produced genetically distinct, authentic HIV-1 viruses to investigate the most obvious sources of laboratory induced error, namely: transfection, RT-PCR and PCR conditions. Using our system we have been able to compare recombination rates between a mix of homozygous viruses (where recombination could only be due to laboratory manipulations) and heterozygous virus (where recombination could be due to laboratory manipulations or to the transfection process). Our data show that the transfection of HEK293T cells with plasmid DNA is an insignificant source of recombination and affirms the appropriateness of using transfection-derived viruses as model systems to identify viral and host cell factors that affect recombination. We stress the importance of using high quality plasmid DNA for the production of virus to minimise the risk of homologous recombination within the culture system. This is consistent with previous studies that have shown that nicked, non-circular plasmid DNA enhances the occurrence of homologous recombination between plasmid and homologous chromosomal regions [8,10].

By comparison considerable attention must be paid to the conditions of RT-PCR and PCR to minimise the occurrence of artefactual chimeras. We postulated that increasing the concentration of RNA during first strand synthesis would decrease the proximal distance of strands of RNA and enhance strand transfer events, thus resulting in increased recombination rates. Our data confirm that RNA concentration in RT-PCR is a critical parameter. Surprisingly, even acceptable ‘mid-range’ concentrations of RNA template (1600 ng, well within the manufacturer’s suggested range) at the RT-PCR step can result in detection of false chimeras. Thus we show that even when using a high fidelity enzyme with low RNase H activity, RNA concentration is a critical factor and can have a marked effect on estimates of recombination rate. We recommend that input RNA be kept to a minimum (100-200 ng) which, in this study, is at least 10-fold lower than that recommended by the manufacturer. In accord with Di Giallonardo et al. [2], we have found that RNaseH activity does not contribute significantly to artificial recombination when the concentration of RNA template is low.

The PCR amplification of DNA is highly susceptible to artefact. Previous studies have shown that input copy number [1,2], annealing and extension steps [1] and number of PCR cycles [1,3] are critical parameters, requiring optimisation to minimise formation of artificial chimeras. In this study we used a 2-step PCR with optimised, limited input copy number (~2500 copies), shown to minimise artificial chimeras [1]. Varying only the number of PCR cycles performed, we have shown that stopping the PCR amplification in the linear phase minimises false recombinants whereas increasing cycle number to 35 dramatically increased the detection of false chimeras. Our data show directly that while increasing the input RNA concentration and PCR cycle might generate a larger viral cDNA population for analysis, such an approach is likely to compromise the quality of sequencing data obtained. We have used the data to develop a protocol that minimises the introduction of artificial recombinants during 454 library generation. The salient features of our protocol are: (i) restricted RNA input (100-200 ng) at RT-PCR for first strand synthesis using a high fidelity RT; (ii) ~2,500 copies of cDNA used as template for subsequent PCR amplification; and (iii) restricting PCR cycles to ~27-29 cycles to ensure that the amplification process is terminated in the linear phase of amplification. These studies have direct relevance to the clinical setting where minimisation of artefact is essential to obtaining accurate measurements of viral diversity and detection of drug resistant mutations. The articulation of these critical parameters (RNA concentration, choice of enzyme, input cDNA copies and PCR cycling parameters) informs our own program of experimental research to identify viral and host cell factors that impact recombination rate and viral diversity.

## Methods

### Molecular clones

**Figure 6:**
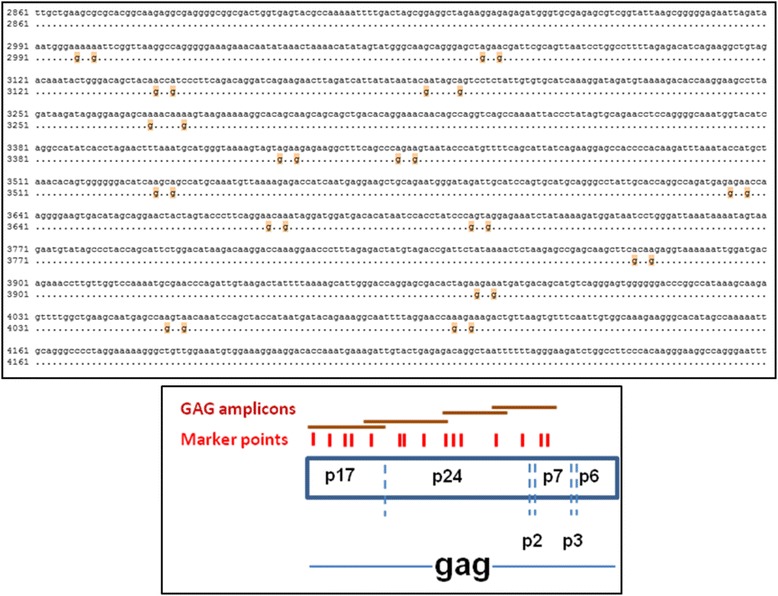
**Measurement of recombination and mutation rates.** (Top) Alignment of GAG1 region of HIV-1 WT sequence (pDRNL(AD8)) (top line) and corresponding marker sequence (bottom line) showing the 15 silent mutations introduced into adjacent codons. This created 14 intervals spaced approximately 50 nucleotides apart (range 18 to 156 nucleotides). Numbering is based on the pDRNL(AD8) WT sequence. Dots indicate identical sequence and silent mutations are indicated with orange highlight. (Bottom) Schematic representation of the marker system depicting silent markers (red) and positions of the overlapping amplicons (brown bars) that enable detection of recombination events (Adapted from [20].

The wild-type (WT) HIV-1 plasmid used was pDRNL(AD8) [19] that encodes an R5-tropic strain of HIV-1. A modified, ‘marker’ version of this plasmid (MK) was created by introducing 15 pairs of silent genetic marker points by nucleotide substitution into *gag*, as previously described [1,20,21] and depicted in Figure 6. This created a total of 13 intervals spaced, on average about 50 nucleotides apart where recombination can be measured. Importantly, the marker points did not change the protein profile of the virus, the replication kinetics compared to wild-type (WT) virus or known RNA sequence elements [1,20,21]. It is important to note that this system does not introduce ‘foreign’ genes into the HIV-1 genome, such as fluorescent markers, antibiotic resistance genes or surface protein markers that can drastically change the RNA structure. Furthermore, the series of silent mutations enables detection of recombination events over a significant length of the genome (Figure 6) and unlike other systems, also permits the identification of multiple template switches. A full description of our system and its attendant advantages in accurately measuring recombination has been published [7,20].

### Transfection and virus production

Homozygous virus was produced by transfection of HEK293T cells with either WT or MK plasmid and heterozygous virus by co-transfection of equimolar amounts of WT and MK plasmids using polyethyleneimine (PEI; Polysciences). Based on Mendelian genetics, the heterozygous virus will be composed of 3 distinct populations: 25% of virus particles will contain 2 strands of WT RNA; 25% will contain 2 strands of MK RNA and 50% will be truly heterozygous, containing a strand of WT and MK RNA. Sixteen to twenty hours after transfection cell cultures were gently washed with PBS and replenished with fresh media. At 48 hr post-transfection viral supernatants were harvested, clarified by centrifugation, layered over a 20% sucrose cushion and centrifuged for 1 hour at 25,500 g, 4oC. Concentrated virus was then further refined by layering onto a sucrose gradient (consisting of 9 sucrose layers ranging from 50% sucrose to 32% sucrose in 2.5% decrements) and centrifuged for 16 h, 25,500 g, 4°C to reduce plasmid contamination. Concentrated virus was resuspended in RPMI (Invitrogen), aliquoted and stored at −80°C. Quantitation of virus was done with an ELISA assay to detect viral capsid protein using the HIV-1 p24^CA^ Antigen Capture Assay (Frederick National Laboratory-AIDS and Cancer virus program).

### RNA isolation and cDNA synthesis

Viral RNA was isolated using TRI-reagent (Qiagen) according to the manufacturer’s recommendations. RNA was reverse transcribed using gene-specific primer, gag4(4195)Rv (5′ ACATTTCCAACAGCCCTTTTTCCTAG 3′), and either SuperScript III reverse transcriptase (RT) (Invitrogen) engineered to have minimal RNaseH activity or AMV RT (with RNAse H activity) (Promega). Variable amounts of input RNA template were used in optimal and ‘stress’ conditions, as described in the results section. To gauge plasmid DNA contamination, RNA samples were processed without reverse transcriptase (RT) and then analysed by qPCR using primers for *gag1* amplicon. In all cases there was a minimum 10 cycle threshold differences between samples processed with the RT and those without, indicating very low levels of plasmid contamination.

### PCR primers

Barcoded PCR primers were designed so that marker points were amplified as 4 overlapping amplicons (~350 bp) covering the *gag* region of HIV-1 and designated *gag1, gag2, gag3 and gag4*. Primers were designed with unique barcodes at the 5′ end (Table 2a) followed by regions that were identical between WT and MK sequence (Table 2b).

**Table 2:** **Primers used for amplicon generation**

**a. Barcode sequences**		
**Barcode**	**Sequence (5′→3′)**	
RL1	ACACGACGACT	
RL2	ACACGTAGTAT	
RL3	ACACTACTCGT	
RL4	ACGACACGTAT	
RL5	ACGAGTAGACT	
RL6	ACGCGTCTAGT	
RL7	ACGTACACACT	
RL8	ACGTACTGTGT	
RL9	ACGTAGATCGT	
RL10	ACTACGTCTCT	
RL11	ACTATACGAGT	
RL12	ACTCGCGTCGT	
RL13	AGACTCGACGT	
RL14	AGTACGAGAGT	
RL15	AGTACTACTAT	
RL16	AGTAGACGTCT	
**b. HIV-1 sequences common to WT and MK transcripts**		
**Primer**	**Sequence**	**Amplicon length (nt)**
G1(2945)Fw	5′ GAGATGGGTGCGAGAGCGTC 3′	370
G1(3314)Rv	5′ TGTGTCAGCTGCTGCTTGCTG 3′	
G2(3236)Fw	5′ ACCAAGGAAGCCTTAGATAAGATAGAGGAAGAG 3′	444
G2(3679)Rv	5′ TGAAGGGTACTAGTAGTTCCTGCTATGTCACTTC 3′	
G3(3584)Fw	5′ GATAGATTGCATCCAGTGCATGCAG 3′	372
G3(3955)Rv	5′ GCTTTTAAAATAGTCTTACAATCTGGGTTCGC 3′	
G4(3793)Fw	5′ TCTGGACATAAGACAAGGACCAAAGG 3′	403
G4(4195)Rv	5′ ACATTTCCAACAGCCCTTTTTCCTAG 3′	

Barcoded primers were designed so that marker points were amplified as 4 overlapping areas within the *gag* region of HIV-1. Primers were designed with (a) unique barcodes at the 5′ end; followed by (b) regions that were identical between both WT and MK cDNA sequence.

### PCR amplification conditions

Modified PCR cycling conditions were used as described previously [1] to reduce chimera formation during PCR amplification. Briefly, the PCR mix (total volume, 15ul) consisted of limiting DNA template (approximately 2,500 copies in 5 ul), 1x HF buffer (Thermo Scientific), 200uM dNTP (NEB), 400nM of each barcoded primer and 0.3U of Phusion DNA polymerase (Thermo Scientific). Five to ten replicates were performed to produce sufficient DNA product for library generation and sequencing. Additionally, to enable monitoring of the reaction using qPCR, duplicates were included containing 0.5x SYBR Green 1 (Life Technologies). PCR cycling conditions were 98°C for 30s, followed by a variable number of cycles of 98°C for 10s and 72°C for 1 min. PCR cycles were selected either to minimise PCR-induced chimera formation (25–29 cycles) or to maximise DNA product and to ‘stress’ PCR conditions (35-40cycles).

### Library generation and 454 sequencing

PCR replicates were pooled and purified using the Wizard SV gel and PCR Clean-Up System (Promega) following the recommended protocol and quantitated against a plasmid standard curve, generated with *gag1* amplicon, ranging from 10^2^-10^6^ copies of DNA per microlitre. Aliquots of each amplicon (*gag1-gag4*) for each sample were pooled to construct sequencing libraries using the 454 library preparation kit (Roche). Emulsion PCR and sequencing were performed using standard XLR70 chemistry at the Institute for Immunology and Infectious Diseases, Perth, Australia.

### Statistical analysis

Sequencing analysis was performed using software custom written in BioRuby. Chimera formation, expressed as recombination rate per 1000 nucleotides (REPN), and statistical comparisons performed as previously described [7]. Recombination is detected by monitoring the linking of marker points in the HIV-1 *gag* gene from WT and MK genomes into a single chimeric genome.
